# The relationship between characteristics of context and research utilization in a pediatric setting

**DOI:** 10.1186/1472-6963-10-168

**Published:** 2010-06-16

**Authors:** Greta G Cummings, Alison M Hutchinson, Shannon D Scott, Peter G Norton, Carole A Estabrooks

**Affiliations:** 1 Faculty of Nursing, 3rd Floor, Clinical Sciences Building, University of Alberta, Alberta, AB, T6G 2G3, Canada; 2 School of Nursing and Midwifery, Faculty of Health Medicine Nursing & Behavioural Sciences, Deakin University, Australia; 3 Cabrini-Deakin Centre for Nursing Research, Cabrini Institute, Cabrini Health, Melbourne, Victoria, Australia; 4 Department of Family Medicine, University of Calgary, UCMC North Hill, 1707, 1632 - 14 Avenue NW, Calgary, AB T2N 1M7, Canada

## Abstract

**Background:**

Research utilization investigators have called for more focused examination of the influence of context on research utilization behaviors. Yet, up until recently, lack of instrumentation to identify and quantify aspects of organizational context that are integral to research use has significantly hampered these efforts. The *Alberta Context Tool *(ACT) was developed to assess the relationships between organizational factors and research utilization by a variety of healthcare professional groups. The purpose of this paper is to present findings from a pilot study using the ACT to elicit pediatric and neonatal healthcare professionals' perceptions of the organizational context in which they work and their use of research to inform practice. Specifically, we report on the relationship between dimensions of context, founded on the Promoting Action on Research Implementation in Health Services (PARIHS) framework, and self-reported research use behavior.

**Methods:**

A cross-sectional survey approach was employed using a version of the ACT, modified specifically for pediatric settings. The survey was administered to nurses working in three pediatric units in Alberta, Canada. Scores for three dimensions of context (culture, leadership and evaluation) were used to categorize respondent data into one of four context groups (high, moderately high, moderately low and low). We then examined the relationships between nurses' self-reported research use and their perceived context.

**Results:**

A 69% response rate was achieved. Statistically significant differences in nurses' perceptions of culture, leadership and evaluation, and self-reported conceptual research use were found across the three units. Differences in instrumental research use across the three groups of nurses by unit were not significant. Higher self-reported instrumental and conceptual research use by all nurses in the sample was associated with more positive perceptions of their context.

**Conclusions:**

Overall, the results of this study lend support to the view that more positive contexts are associated with higher reports of research use in practice. These findings have implications for organizational endeavors to promote evidence-informed practice and maximize the quality of care. Importantly, these findings can be used to guide the development of interventions to target modifiable characteristics of organizational context that are influential in shaping research use behavior.

## Background

In 2006 and 2007 respectively, special issues of the *Journal of Continuing Education in the Health Professions *(JCEHP) and *Nursing Research *were dedicated to advancing the theory and science of knowledge translation and research utilization by the healthcare professions with the ultimate aim of improving health outcomes. The JCEHP issue emphasized that the transfer and uptake of research findings in healthcare settings remains slow and unpredictable, despite considerable investment of public funds into healthcare research,[1] describing numerous theoretical approaches to how knowledge transfer could be supported [2]. The *Nursing Research *special issue advanced the science of KT by using the *Promoting Action Research in Health Services (PARiHS) *framework [3] to frame the analysis of relationships between various dimensions of organization context in hospitals and nurses' reported research use [4]. In contexts characterized by better leadership, empowering work environments (culture), and open feedback on performance (evaluation), nurses reported significantly greater research use [5], which was related to better patient outcomes (reduced adverse patient events) [6]. In summary, research to date suggests the need for a parsimonious, valid and reliable measure of healthcare context, which was met by the development and assessment of the Alberta Context Tool (ACT) [7].

## Aim

The aim of this paper is to report findings from a pilot study assessing the ACT, an instrument designed to measure modifiable dimensions of organizational context and self-reported research utilization. Specifically, we describe the relationship between three dimensions of context (culture, leadership and evaluation) as defined by the *Promoting Action on Research Implementation in Health Services *(PARiHS) framework[3], and research utilization behavior in neonatal and pediatric settings.

## Literature

### Promoting Action on Research Implementation in Health Services

Since its first publication in 1998, the PARiHS framework [3] of research implementation has gained increasing attention. In this framework, three constructs are considered essential for the successful implementation of research into practice: evidence, facilitation, and context. Evidence includes both codified and non-codified sources of knowledge including research evidence, clinical expertise, local data or information, and patient experience. An underlying assumption of the conceptualization of evidence is that these different evidence forms are melded, through negotiation and shared understanding, within a complex and multifaceted clinical environment [8]. Context is understood to be "the environment or setting in which the proposed change is to be implemented" [[3], p. 150]. Context is proposed to have three dimensions (culture, leadership and evaluation). Early PARiHS development work suggested that research implementation would be facilitated by a value-oriented culture that is receptive to change; clear, transformational leadership that supports teamwork and staff involvement in decision making; and multiple methods of evaluation at various levels [9]. The final element, facilitation, involves "providing help and support to achieve a specific goal to enabling individuals and teams to analyze, reflect and change their own attitudes, behaviors and ways of working" [[10], p. 580].

### Research Utilization

Research utilization, a specific form of knowledge utilization, is a complex process in which research findings are transformed from one or more studies into instrumental, conceptual or persuasive utilization [11]. *Instrumental *research utilization is the direct or concrete application of research findings, often identified as the application of clinical practice guidelines, procedures and clinical protocols. *Conceptual *research utilization occurs when research serves an 'enlightenment' function [12,13]: that is, practitioners' become aware of research findings, and the findings inform, broaden or alter their thinking and practice in indirect ways [11]. *Persuasive *or *symbolic *research utilization is when research findings are used as a tool to advocate for a certain procedure or practice. Generally speaking, overall research utilization can be defined as the use of research findings in any and all aspects of one's work [14].

Poor uptake of research in clinical practice has largely been attributed to individual characteristics such as a practitioner's inability to understand research (a lack of research skills and inadequate educational preparation), age, or attitude toward research [15-17]. Consequently, the majority of the research in the field has centred on trying to understand individual level barriers and facilitators to research utilization [18-22]. Much of the work in nursing has been in this vein and has explored individual determinants of research use [23] with the predominance of studies employing bivariate statistical approaches. These approaches do not permit interactional or causal exploration. Furthermore, syntheses of findings of studies on the individual determinants of research utilization to date are equivocal, and the majority of those currently studied (e.g., age, years of clinical experience) are questionably modifiable [23].

### A Shift to Exploring Organizational Factors

Previous work demonstrates that even if clinicians have adequate and recent research-based knowledge they do not automatically use it in practice [24]. There is growing consensus that the challenges in transferring research into clinical practice are often more due to organizational factors than to attributes of individual clinicians or the methods by which research findings are disseminated [16,18,25]. Given that the majority of healthcare professionals work in complex organizational environments, redirecting research efforts toward understanding the influence of organizational context is warranted. Organizational context is believed to shape the utilization of research in practice [26] through its influence on individual and group behavioural norms and innovation.

Despite organizational context being consistently identified as an important factor influencing nurses' use of research, it has not been well studied [23,27]. Kitson and colleagues [3,9,28] have begun to investigate more thoroughly the importance of organizational features (e.g., culture, leadership) in influencing research use. Despite a lack of research on how organizational context or work environment influences research utilization, some recent investigations have begun to shed light on the nature of and processes inherent in organizational context. Using qualitative methods Scott and colleagues [29] found that uncertainty within an organizational context (e.g., inconsistent management) significantly hindered nurses' use of research in practice. Investigators have successfully identified contextual features that influence research utilization and more sophisticated analytic work including the development and testing of models to demonstrate how these features work and interact, have started to emerge. For instance, in a study underpinned by the PARIHS framework, Wallin and colleagues [30] used multivariate procedures to derive a score of nurses' research utilization and then demonstrate that degrees of context from low to high were significantly related to increasing research utilization scores. Subsequently, Cummings and colleagues [6] used structural equation modeling to test a theoretical model of relationships among features of organizational context (e.g. responsive administration, relational capital, and hospital size), nurses' research utilization scores and adverse patient events. They found that these organizational characteristics interacted with better leadership, culture, and evaluation, to lead to reports of greater research use by nurses, which then led to fewer adverse patient events. These findings suggest that strategies to improve dimensions of organizational context could potentially increase research utilization behaviors. The haphazard nature of research utilization is frequently attributed to the unique contexts in which research is implemented. Further sophisticated analytic work is needed in order to make theoretical advancements and to identify contextual predictors of research use.

### Research Utilization in Child Health

Child healthcare settings are not immune to the challenges of applying the best available research evidence in clinical practice. Thus, ensuring research is used to inform clinical practice is of central importance. Effective research utilization is fundamental to ensuring that the best available research evidence informs the health and healthcare of infants, children, youth and families. Research shows that provision of research-informed care to children not only improves health outcomes, but also reduces healthcare utilization [31-35]. Yet, studies of adult populations in the USA and the Netherlands suggest that 30-40% of patients do not receive care that is well supported by scientific evidence, and 20-25% of their care is either not needed or potentially harmful [36,37]. Similar findings have been demonstrated in Canadian child health research [38].

To date, information about research utilization among child healthcare professionals [39] is lacking. In fact there is growing recognition that health services research in child health has been largely under-represented [40]. Understanding the patterns of research utilization in child health contexts is an important first step to help address these gaps.

## Methods

We used a cross-sectional survey design to elicit health professionals' opinions about aspects of the organizational context in which they work and the extent to which they use research evidence to inform their practice.

### Sample and setting

Nurses, managers, clinical specialists, doctors and allied healthcare professionals working in one of three pediatric units (one pediatric inpatient unit and two pediatric/neonatal critical care units) located in two university affiliated hospitals in Alberta, Canada were invited to participate in this study. For this study we analyzed data only from the nursing sample.

### Measures

The Alberta Context Tool (ACT), conceptually framed by the PARiHS framework, was used to collect the data. The index version of the ACT was developed for use in acute care settings and comprises a suite of survey instruments designed to assess modifiable characteristics of organizational context and self-reported research use [41]. The refined ACT consists of 56 items reflecting the following eight contextual dimensions: culture, leadership, evaluation, social capital, informal interactions, formal interactions, structural and electronic resources, and organizational slack (representing three sub-concepts - time, space, human resources). Each dimension is measured by a separate scale or set of items. For the culture, leadership and evaluation scales, each item in the instrument used a five-point Likert-type scale (1 = strongly disagree, 2 = disagree, 3 = neither agree nor disagree, 4 = agree, 5 = strongly agree). In this study, we used data from the three PARiHS contextual dimensions (culture, leadership and evaluation) due to the smaller sample size obtained in this pilot study and to replicate Cummings et al's [6] approach to differentiating contexts from high to low. Additionally we hypothesized that there were positive relationships between the combined core contextual factors of culture, leadership and evaluation and nurses' research utilization.

Finally, measures of instrumental and conceptual research utilization, the dependent variables in this study were developed and validated by Estabrooks [14,30,42,43] and used in conjunction with the ACT in this pilot study. Four single items (not combined to form an index) measured four kinds of research use: instrumental, conceptual, persuasive, and overall. Each item was preceded by a definition and examples of that kind of research use. For each kind of research use, respondents were asked to indicate how often they used research in this way on their last typical workday. Items were scored on a 5-point scale (from 10% or less to almost 100%). In this study we used conceptual and instrumental research utilization as our dependent variables due to the small sample and because nurses have identified that these are more directly linked to their practice [44].

The ACT was pilot tested in adult acute care settings and factor analytic procedures demonstrated that almost 70% of the variance in the context construct was accounted for in a sample of nurses, clinical specialists, managers, doctors and allied healthcare professionals working in four major teaching hospitals [41]. The instrument was further refined based on psychometric and bivariate analysis of these data. The resulting instrument contained 56 items and was completed in approximately 9 minutes. Principal Components Analysis produced a 13-factor solution that accounted for 59.26% of the variance in perceptions of organizational context. Acceptable internal reliability for the culture, leadership and evaluation dimensions of organizational context was found with Cronbach's alpha scores of .72, .91 and .91 respectively [7]. Additional detail on group alphas and other psychometrics of the ACT are described elsewhere [45].

For the current study some adaptation was made to the ACT to ensure the language and content were appropriate to the pediatric setting. Specifically, the word *patient *was substituted with *patients and families*; the examples which preceded questions were revised to ensure relevance to the pediatric setting. The culture, leadership and evaluation dimensions of organizational context were again found to be reliable with Cronbach's alpha scores of .71, .90 and .87 respectively [45].

### Ethical approval

Ethical approval to conduct the study was received from the University of Alberta Health Research Ethics Board. Operational and administrative approval to conduct the study was also attained. The surveys were completed anonymously and confidentiality of the data was maintained.

### Recruitment

We used a modified Dillman [46] approach to recruit survey respondents (reported in detail elsewhere [47]). The recruitment procedure was initiated four months before the data collection commenced, and involved a series of formal presentations and informal (one-on-one) interactions with staff to familiarize them with study aims and processes of data collection. In particular, the presentations included information about use of the Internet to access and complete the instrument. Additionally, electronic and print materials were used to promote awareness of and communicate information about the study. On a weekly basis, we distributed colorful posters to report graphically the cumulative response rate for each unit. This strategy increased the study profile and prompted potential respondents to complete the survey.

### Data collection

This study was undertaken in 2007. Data were collected using paper-based and electronic survey methods. University of Alberta Population Research Laboratory (PRL) http://www.uofaweb.ualberta.ca/prl/index.cfm was contracted to prepare and administer the electronic and paper-based surveys. All healthcare professionals who were eligible to complete the survey were provided with a personalized survey package. The package contained a letter to introduce the study, a business card providing the Uniform Resource Locator (URL) for the survey and a unique password, information detailing how to complete the survey online, a signed continuing education certificate, and a five-dollar gift certificate. PRL assembled the packages, which were either hand delivered to each eligible staff member on the three units or placed in their work setting mailboxes. Consistent with a modified Dillman [46] approach, all eligible participants were sent a postcard reminder two weeks and four weeks after commencement of data collection. The non responding nurses in the sample were also sent a paper copy of the survey with this final reminder.

### Analysis

Drawing on the approach used by Wallin et al. [30] and Cummings et al. [6], we used respondents' scores on the three context dimensions (culture, leadership and evaluation) to sort data into four conceptually distinct contextual groups (high, moderately high, moderately low, and low) within their unit. To preserve the sample size for this pilot study, mean scale scores were calculated for all individuals who responded to at least two items in each scale. If respondents only answered one question in a scale, that score was used. This allowing us to retain another 83 cases (23%) of the respondents, as 275 respondents had no missing data on the three context scales. Then analysis of variance was used to compare the culture, leadership, evaluation and research use scores across the three units.

In order to sort nurses into the four contextual groups, we needed to effectively divide the number of respondents into high and low on each dimension and therefore required an appropriate cut point between high and low mean scores on each of the 5-point leadership, culture and evaluation scales. In our earlier work [6,30] the 4-point Likert scale from Strongly Agree to Strongly Disagree made the choice of cut point simple - nurses who had reported Strongly Agree and Agree for a contextual dimension were categorized as high, and those reporting Strongly Disagree and Disagree were categorized as low. In our current study, the 5-point Likert included a 3 = neither agree nor disagree between 4 = Agree and 2 = Disagree. Therefore we examined three possible cut points for nurses' individual means on the contextual dimensions; 3.0, 3.5, and 3.9. Both 3.0 and 3.9 provided skewed groupings where insufficient numbers of nurses were found in low groups for 3.0 and in high groups for 3.9. On this basis we selected 3.5 as the cut-off score to categorize contextual dimensions as 'high' (an individual respondent's mean score > 3.5) or 'low' (an individual respondent's mean score ≤ 3.5). Respondents' data were grouped using their mean scores of the three contextual scales (culture, leadership and evaluation) to create four distinct groups. Hence, those who reported a high score on all three organizational context scales were categorized as working in a *high context*. Those who reported a high score on any two context variables and a low score on any single variable were categorized as working in a *moderately high context*. Those who scored high on any single variable and low on the two remaining variables were categorized as working in a *moderately low context*. Finally, those who scored low on all three context variables were categorized as working in a *low context*. Grouping the respondents in this manner enabled us to examine their self-reported research use behaviors in relation to their perceptions of organizational context.

## Results

The instrument was offered to 362 nurses, including registered, graduate and licensed practical nurses. A response rate of 69% was achieved for a total sample size of 248. Internal reliability for the culture, leadership and evaluation scales was acceptable with Cronbach's alpha scores of .688, .898, .849 respectively.

The demographic characteristics of the nurses from each of the three units are reported in Table 1. Approximately 96% of the nurse respondents were female. The highest level of education reported was a Master's degree (N = 1, 0.4% of the total sample); the majority of nurse respondents (N = 154, 61.4% of the total sample) reported having obtained a Bachelor's degree. Across the units the proportion of nurses who reported having a Diploma/certificate was approximately equal. Similarly, the proportion of nurses who possessed a Bachelor's degree was similar in each of the units. The mean number of years the nurses were employed in their current position was 10.1 (SD 9.6). For units B and C the mean number of years in the current position was 11.03 (SD 9.06) and 11.58 (SD 10.27), respectively. This contrasted with an average of 5.79 (SD 7.5) years of work in their current position reported by nurses in Unit A.

**Table 1:** Sample Demographics

		Unit A	Unit B	Unit C	Total (%)
**N (%)**		55 (22.2)	68 (27.4)	125 (50.4)	248
**Gender** **[N, (%)]**	**Male**	4 (7.3)	3 (4.4)	0	7 (2.8)
	**Female**	51 (92.7)	63 (92.6)	124 (99.2)	238 (95.9)
	***Missing Values***	*0*	*2 *(2.9)	*1 *(0.8)	*3 (1.2)*
**Education** [N, (%)]*	**Diploma/Certificate**	26 (47.3)	32 (47.0)	62 (49.6)	120 (48.4)
	**Bachelors Degree**	35 (63.6)	43 (63.2)	76 (60.8)	154 (61.4)
	**Masters Degree**	0	0	1 (0.8)	1 (0.4)
	**Missing Values**	0	0	0	0
**Years in current position** **[mean (SD)]**		5.79 (7.5)	11.03 (9.06)	11.58 (10.27)	10.1 (9.6)

* Respondents were asked to identify all that apply, and therefore the numbers are larger than the study n.

Table 2 presents the results of analysis of variance of mean unit scores for culture, leadership, evaluation and research use. Statistically significant differences were found between groups for perceptions of culture, leadership and evaluation, and for self-reported conceptual research use, while no significant differences were found in instrumental research use between the groups. On average, nurses working on Unit A were the most positive group in their perceptions of culture and leadership. Nurses working on Unit B were the most positive in their perceptions of evaluation.

**Table 2:** Contextual Dimensions and Research Use Descriptives

	Mean (SD) (N)				ANOVA	
	
	All Nurses	Unit A	Unit B	Unit C	F-Statistic	p-value
**Culture**	3.49 (0.65) (N = 248)	3.88 (.54) (N = 55)	3.25 (.69) (N = 68)	3.46 (.61) (N = 125)	16.484	.000
**Leadership**	3.51 (0.79) (N = 248)	3.97 (.80) (N = 55)	3.22 (.81) (N = 68)	3.46 (.68) (N = 125)	15.767	.000
**Evaluation**	3.31 (0.72) (N = 246)	3.12 (.80) (N = 53)	3.61 (.64) (N = 68)	3.23 (.68) (N = 125)	9.410	.000
**Instrumental Research Use**	3.28 (1.35) (N = 225)	2.94 (1.32) (N = 54)	3.30 (1.36) (N = 66)	3.45 (1.34) (N = 105)	2.510	.084
**Conceptual Research Use**	3.55 (1.26) (N = 241)	3.36 (1.21) (N = 53)	3.03 (1.38) (N = 66)	3.91 (1.10) (N = 122)	12.227	.000

Table 3 presents mean scores and standard deviations for instrumental and conceptual research use for all nurses in the sample, by unit, and by category of context. Higher self-reported instrumental and conceptual research use by all nurses in the sample was associated with more positive perceptions of organizational context. For Unit B and when data for nurses from all three units were combined, self-reported instrumental use of research is consistently higher in relation to more positive perceptions of organizational context. Whereas nurses on Unit A who perceived their context as 'high' reported slightly lower instrumental research use than those who reported context as 'moderately high'. Nurses on Unit C, who perceived the organizational context as 'moderately low', reported higher instrumental research use when compared with their colleagues who perceived their context as 'moderately high'. However, nurses who perceived the organizational context as 'high' clearly reported the highest instrumental use of research for Unit C.

**Table 3:** Instrumental and conceptual research use by context category for each unit

Context	All Nurses [mean (SD)] (N)		Unit A [mean (SD)] (N)		Unit B [mean (SD)] (N)		Unit C [mean (SD)] (N)	
	Instrumental research use	Conceptual research use	Instrumental research use	Conceptual research use	Instrumental research use	Conceptual research use	Instrumental research use	Conceptual research use
**High**	3.69 (1.26) (N = 48)	4.12 (.79) (N = 51)	3.07 (1.27) (N = 14)	4.07 (.73) (N = 14)	3.80 (1.48) (N = 10)	3.90 (.88) (N = 10)	4.00 (1.06) (N = 24)	4.22 (.80) (N = 27)
**Moderately high**	3.39 (1.32) (N = 70)	3.45 (1.26) (N = 71)	3.12 (1.31) (N = 26)	3.24 (1.23) (N = 25)	3.65 (1.04) (N = 20)	2.95 (1.05) (N = 20)	3.46 (1.53) (N = 24)	4.04 (1.25) (N = 26)
**Moderately low**	3.29 (1.30) (N = 58)	3.36 (1.42) (N = 66)	2.75 (.96) (N = 4)	3.00 (1.15) (N = 4)	3.09 (1.35) (N = 23)	2.67 (1.52) (N = 24)	3.52 (1.29) (N = 31)	3.84 (1.20) (N = 38)
**Low**	2.72 (1.38) (N = 47)	3.37 (1.30) (N = 51)	2.25 (1.39) (N = 8)	2.75 (1.49) (N = 8)	2.77 (1.59) (N = 13)	3.17 (1.70) (N = 12)	2.85 (1.29) (N = 26)	3.61 (1.02) (N = 31)

The pattern for conceptual research use and organizational context categories was slightly different. While nurses on Units A and C reported steadily higher conceptual use of research in conjunction with more positive perceptions of the organizational context; conceptual research use reported by nurses on Unit B, and for *all *nurses combined, did not follow this pattern. For *all *nurses combined, those reporting the organizational context as 'low' reported fractionally higher conceptual use of research than did nurses perceiving the organizational context as 'moderately low'. On Unit B, nurses who perceived the organizational context as 'low' reported higher conceptual research use than did their colleagues who perceived the organizational context as 'moderately low' or 'moderately high'.

The appropriateness of the unit of analysis at individual or unit levels for research use was assessed using interclass correlations[48]. Table 4 depicts the ICC(1) scores which indicate that instrumental research use is best analyzed at the individual level (all ICC(1) scores being less than .10). Two ICC(1) scores for conceptual research use (CRU) in moderately high and low contexts suggest that CRU could be analyzed at the unit level, yet the scores for high and low contexts suggest individual unit of analysis is most appropriate. Our earlier analyses of ICC of the ACT contextual dimensions [41,45] support their aggregation to the unit level.

**Table 4:** Aggregation at Unit level by Context category

Context Category	Variable	F	BMS	WMS	ICC1	ICC2	eta_2	Omega_2	p-value
**Low**	IRU	0.5712	1.1060	1.9362	0.0000	0.0000	0.0253	0.0000	0.5690
	
	CRU	1.6505	2.7000	1.6359	0.0425	0.3941	0.0643	0.0249	0.2027

**Moderately Low**	IRU	1.8964	3.2092	1.6923	0.0446	0.4727	0.0634	0.0295	0.1596
	
	CRU	5.8959	10.2428	1.7373	0.2032	0.8304	0.1556	0.1275	0.0045

**Moderately High**	IRU	0.9789	1.7118	1.7487	0.0000	0.0000	0.0284	0.0000	0.3810
	
	CRU	5.3239	7.5530	1.4187	0.1536	0.8122	0.1354	0.1086	0.0071

**High**	IRU	2.6325	3.8920	1.4784	0.0929	0.6201	0.1047	0.0637	0.0829
	
	CRU	0.6287	0.3994	0.6353	0.0000	0.0000	0.0255	0.0000	0.5376

**Figure 1:**
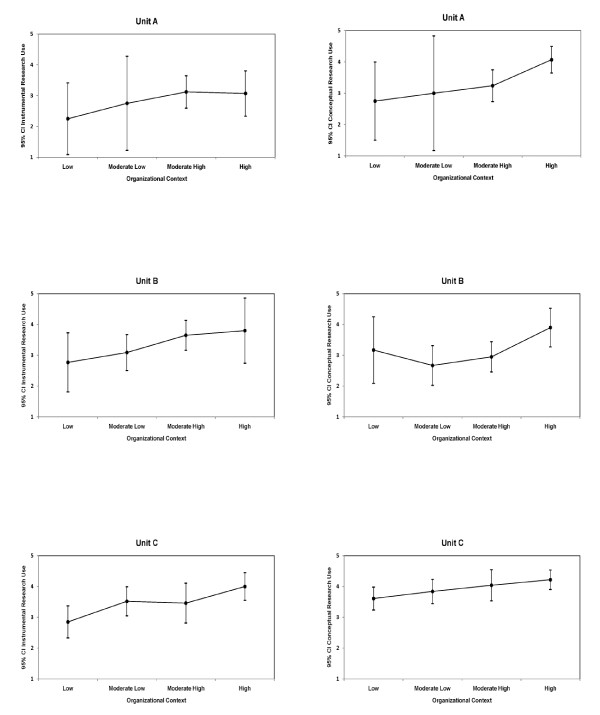
**Nurses' instrumental and conceptual research use according to context category, by unit**.

Figure 1 illustrates for each unit, the patterns described above for nurses' self-reported instrumental and conceptual research use along with the 95% confidence interval according to the four context categories. For both instrumental and conceptual research use, a general pattern of increasing degrees of research use was associated with more positive perceptions of context across all three units. The highest levels were associated with high perceptions of context across all units with the exception instrumental research use in Unit A as previously described.

## Discussion

These data illustrate a positive trend in the relationships between organizational context and research utilization. That is, more positive perceptions of organizational context were generally associated with higher self-reported instrumental and conceptual research use. We have organized our discussion around three key points: 1) the nature of the relationship between instrumental research use and organizational context, 2) new insights into the potential appropriate 'level' of analysis (individual or unit) of the concepts studied (research use, culture, leadership and evaluation), and 3) the establishment of a methodological 'cut off' point for distinguishing between 'low' and 'high' contexts.

First, our findings are consistent with Wallin et al. [30] and Cummings et al. [6] who studied nurses from adult acute and general hospitals only, in that, nurses who worked in high levels of contexts reported higher levels of instrumental research use. Thus, these findings support our initial claims that in pediatric environments, context shapes research utilization behaviors. In sum, nurses working in contexts characterized by strong leadership, positive feedback and culture reported more instrumental and conceptual research utilization than nurses working in less positive contexts. This study also adds to the work of Wallin et al. [30] and Cummings et al. [6] by using specific questions to directly measure instrumental and conceptual research utilization, in contrast to a derived research utilization score. Thus to some degree these findings also add some level of justification to the derivation procedures for the outcome variable (research utilization) in those earlier studies.

Our findings provide further empirical support for the context dimension of the PARiHS model [3,9,28]. Specifically, both instrumental and conceptual research utilization scores were higher in association with more positive contextual conditions (culture, leadership and evaluation). Yet, these relationships are stronger for instrumental research use. Although caution must be used in light of the relatively small sample size, we believe based on this observation in this pilot study that the influence of culture, leadership and evaluation may align more closely with instrumental research use. It is possible that the questions contained within these scales are more congruent with the act of 'doing' and, therefore, with instrumental use of research. As well, these findings imply that through concerted efforts to improve culture, leadership and evaluation, higher degrees of research utilization may be achievable. Instrumental research utilization directly relates to "doing" things, that is, providing direct, observable action associated with the application of research findings. In today's healthcare environments, doing is valued and nurses are expected to complete tasks at work [26,49]. As a result, it makes sense that acute care pediatric nurses would see more direct relevance, in terms of their work, for instrumental research utilization. Additionally, nurses may more readily recall and report instances of instrumental research use than other more covert forms of research use.

Second, our findings using the three contextual dimensions aggregated to the unit level suggest that they behave appropriately as unit level variables - both by the near linear trends in relationships with research use and the consistently small standard deviations across units (Table 2). Our aggregation analyses reported in Table 4 and the relatively larger standard deviations of both instrumental and conceptual research use across individual nurses within a unit, seen in Table 2 suggest that these variables are appropriately analyzed at the individual level. Conceptually, this assertion aligns with Estabrooks' research [50]; that is, research utilization behaviors are individual level phenomena that can then be shaped by the context in which one works. However, these findings suggest that more targeted methodological work needs to be completed in order to determine whether research utilization scores from individual participants can be 'averaged' across groups of individuals working in a similar context and still remain meaningful.

Third, the results of this pilot study lay the foundation for future studies. Literature on dichotomizing continuous variables suggests the importance of establishing cut points because prior work can be invalidated if cut points change [51]. We established a methodological 'cut off' point for distinguishing between 'low' and 'high' contexts. This pilot work sets the foundation for future analyses in a variety of settings and professional groups, each of which will provide opportunities for validation and reliability procedures. We did not want to use the median score on each contextual dimension as it suggests relativity in scores across studies, disciplines and settings. Establishing an appropriate cut point for analyzing high and low aspects of contextual dimensions is an important study outcome [52].

## Limitations

The sample is drawn from university hospitals only and these hospitals are linked operationally. The sample includes healthcare professionals from pediatric intensive care units and therefore the results cannot be generalized beyond this setting. This was a pilot study hence the sample size is small and this has restricted the degree of analysis that could be undertaken.

## Conclusion

Overall, the results of this study lend support to the claim that more positive organizational contexts influence greater use of research in practice in child health settings. These findings have important implications for organizational endeavors to promote evidence-informed practice and maximize the quality of care. Importantly, these findings can be used to guide the development of interventions to target modifiable characteristics of child health organizational contexts that are influential in shaping research use behavior.

## Competing interests

The authors declare that they have no competing interests.

## Authors' contributions

GGC led the design and interpretation of statistical analysis and development of the manuscript. AH contributed to the interpretation of statistical analysis and manuscript development.

SS contributed to the design and conduct of the study as the Alberta site investigator, and through interpretation of statistical analysis and manuscript development. PN contributed to manuscript development. CAE is the Alberta lead investigator and as such secured resources, led the design and conduct of the study and provided guidance on manuscript development. All authors approved the final manuscript.

## Pre-publication history

The pre-publication history for this paper can be accessed here:

http://www.biomedcentral.com/1472-6963/10/168/prepub
